# SOCIOEMOTIONAL FACTORS DRIVING SEXUAL INTEREST AND SATISFACTION IN OLDER ADULTHOOD

**DOI:** 10.1093/geroni/igae098.3722

**Published:** 2024-12-31

**Authors:** Annie Nguyen, Malinee Neelamegam, Matthew Smith

**Affiliations:** University of California San Diego, La Jolla, California, United States; University of North Texas Health Science Campus, Fort Worth, Texas, United States; Texas A&M University, College Station, Texas, United States

## Abstract

Research shows that sexual interest declines with age, while sexual satisfaction remains relatively stable in later life. Declines in sexual interest can result from health deficits that pose barriers to sex; however, for older adults who are healthy and able to have sex, socioemotional factors may influence sexual satisfaction. This study examined the associations between socioemotional variables, sexual interest, and sexual satisfaction among older adults. Data were drawn from the subset of adults ages 60+ in UCSD SAGE study (n=359). A series of linear regression models were fitted to examine sexual interest and sexual satisfaction as separate outcomes while accounting for demographics (age, gender, marital status, race/ethnicity), socioemotional variables (morale, self-compassion, satisfaction with life), and health indicators (physical function, general health). Older adults who were younger ages (B=-.172, p=.001), men compared to women (B=.283, p<.001), married/partnered compared to single (B=.213, p<.001), and had better physical functioning (B=.214, p<.001) reported higher sexual interest. In the model for sexual satisfaction among sexually active older adults (n=148), those with greater morale (B=-.027, p=.03) and better physical function (B=.271, p=.003) reported higher sexual satisfaction. Findings reinforce the importance of physical function for sexual interest and satisfaction among older adults. Socioemotional factors, such as morale, are more strongly associated with sexual satisfaction. However, these relationships may be bidirectional or recursive in nature (i.e., those with higher morale are satisfied with sexual encounters, and that sexual satisfaction may lead to higher morale), requiring additional longitudinal studies.

